# DUAL-tDCS Treatment over the Temporo-Parietal Cortex Enhances Writing Skills: First Evidence from Chronic Post-Stroke Aphasia

**DOI:** 10.3390/life11040343

**Published:** 2021-04-14

**Authors:** Francesca Pisano, Carlo Caltagirone, Chiara Incoccia, Paola Marangolo

**Affiliations:** 1 Department of Humanities Studies, University Federico II, 80133 Naples, Italy; francescapisano00@virgilio.it; 2 IRCCS Santa Lucia Foundation, 00179 Rome, Italy; c.caltagirone@hsantalucia.it (C.C.); c.incoccia@hsantalucia.it (C.I.)

**Keywords:** post-stroke aphasia, tDCS, temporo-parietal-cortex, writing abilities, language recovery, sublexical route treatment

## Abstract

The learning of writing skills involves the re-engagement of previously established independent procedures. Indeed, the writing deficit an adult may acquire after left hemispheric brain injury is caused by either an impairment to the lexical route, which processes words as a whole, to the sublexical procedure based on phoneme-to-grapheme conversion rules, or to both procedures. To date, several approaches have been proposed for writing disorders, among which, interventions aimed at restoring the sub-lexical procedure were successful in cases of severe agraphia. In a randomized double-blind crossover design, fourteen chronic Italian post-stroke aphasics underwent dual transcranial direct current stimulation (tDCS) (20 min, 2 mA) with anodal and cathodal current simultaneously placed over the left and right temporo-parietal cortex, respectively. Two different conditions were considered: (1) real, and (2) sham, while performing a writing task. Each experimental condition was performed for ten workdays over two weeks. After real stimulation, a greater amelioration in writing with respect to the sham was found. Relevantly, these effects generalized to different language tasks not directly treated. This evidence suggests, for the first time, that dual tDCS associated with training is efficacious for severe agraphia. Our results confirm the critical role of the temporo-parietal cortex in writing skills.

## 1. Introduction

Aphasia is an acquired language impairment following left-hemisphere brain injury [1]. The aphasic symptoms vary in terms of severity and degree of involvement across the different language modalities, such as oral expression, comprehension, reading, and writing. Despite the fact that clinicians and therapists are generally more attentive to spoken than written language disorders, persons with aphasia (PWA) also show severe difficulties in writing [2,3], which interferes with everyday activities (e.g., to take notes; to make a shopping list). Indeed, to date, due to the overuse of internet devices (i.e., computers, tablets, mobile phones, emails), written language has become more important than previously considered.

One of the major models proposed for writing is the dual-route model (DRM). In its most simplified version, the model assumes two independent procedures which operate in parallel: a lexical route which processes words as a whole, and a sublexical one based on phoneme-to-grapheme conversion procedures [4,5,6]. A dual-route model accounts for how a literate person can write both regular, irregular words and legal nonwords. As within the lexical route for word naming, the lexical route for writing includes two stores encompassing the phonological and orthographic lexical representation of words and a semantic store which contains their semantic representation [7]. This route allows a person to write any type of familiar words (regular vs. irregular) but cannot be used when spelling unfamiliar words or nonwords; thus, it is the only method available for writing irregular words [4,5,6]. On the contrary, sublexical procedures rely on phoneme-to-grapheme conversion rules which translate a string of sounds (i.e., phonemes) into its corresponding graphemes. This procedure is used to write regular words and nonlexical phonemic strings (nonwords) [4,5,6,8].

The major evidence for a dual-route procedure for writing derives from the observation of PWA affected by writing disorders [9,10,11,12,13]. Indeed, the writing deficits an adult subject may acquire after left hemispheric brain injury might be caused by either an impairment to the lexical route, to the sublexical one or to both procedures. The most frequent syndrome due to a damage to the lexical pathway is surface dysgraphia [9,14]. Errors in spelling irregular words are the most frequent symptom. Thus, this syndrome is more easily detected in languages with irregular spelling such as English. In “transparent languages” (i.e., Italian, Spanish), this difficulty translates into “dysorthography” [8,15,16,17]. Indeed, in those languages, the sub-lexical route allows a person to correctly transcribe the phonological strings through the phoneme-to-grapheme correspondence based on either sound–letter conversion or syllabic conversion, so that the lexical route is largely superfluous [8,17,18]. The alternate pattern of impairment, due to damage to the phoneme-to-grapheme conversion procedure, leads to phonological dysgraphia. In this case, if the word is common and stored in the orthographic lexicon, the word may still be spelled appropriately, while, if the word is unknown, writing errors would occur [10,19]. Thus, patients with selective damage to the phonological pathway may still be able to write both regular and irregular familiar words, but they cannot write unfamiliar words or stimuli that are not real words (nonwords) which rely on the sub-lexical route [10,19]. In the most severe cases, both the lexical and the sublexical routes are damaged, resulting in central agraphia—the complete loss of the ability to communicate through writing [20].

To date, several rehabilitative approaches have been proposed for writing disorders which aim either at restoring the compromised written subcomponents or at promoting compensatory strategies [3,21,22,23]. Treatments targeting sub-lexical processes in writing require the patient to segment the words and/or nonwords into syllables and phonemes, to write graphemes for each dictated phoneme and to associate a specific grapheme with the words starting with that grapheme [3,6,24,25,26]. In the case of Italian, specific training aimed at restoring the sub-lexical route was also successful in cases of severe agraphia since, due to the transparency of the language, this procedure also offers a rapid generalization of the acquired learning to untrained items [27]. Indeed, generalization to untreated items is expected as, through this procedure, the patient learns the correspondence between sounds and graphemes regardless of their position within the word. Since in the Italian language the conversion procedures take place at the syllabic level [17,28,29,30], syllabic segments were used to stimulate the sublexical processes. Accordingly, from a development point of view, in the early phases of writing acquisition, Italian children segment the phonological input string and translate into the corresponding orthographic sequence. Later on, after a rapid development of the sub-lexical route, they gradually acquire the orthographic lexical representation of the whole word, relying on the lexical route [17,28,29,30].

To date, new treatment approaches have emphasized the role of non-invasive brain stimulation techniques, such as transcranial direct current stimulation (tDCS), in enhancing language improvement in aphasic individuals (for a review, see [31]). Through tDCS, a weak electrical current (1–2 mA) is administered via two surface electrodes applied to the scalp. It is generally assumed that anodal stimulation increases the excitability over the targeted area, while cathodal stimulation diminishes it by affecting the resting membrane potential of the cell [32]. Depending on the duration, polarity and intensity of stimulation, these effects may last for minutes to hours compared with a placebo condition (known as a “sham” condition), in which the stimulator is turned off after 30 s [33,34]. More recently, a new approach, “Dual-tDCS”, has been suggested, in which the left and the right hemisphere are simultaneously stimulated with opposite current. In the case of aphasic disorders, generally, anodal stimulation has been delivered over the left injured language areas with cathodal stimulation over the right homologous ones [35,36,37,38]. Indeed, although several studies have shown that the chronically reorganized language system can sometimes engage homotopic language areas in the right hemisphere [39,40,41], particularly in the case of an extended lesion to the left hemisphere [40,42,43], an abnormal interhemispheric imbalance due to an increase in the excitability of the undamaged right hemisphere which exerts interhemispheric inhibition (IHI) over the lesioned one has also been often described after unilateral left-hemispheric stroke. Thus, in order to restore this maladaptive condition, dual-tDCS has also been proposed (see for a review [31,44]). To our knowledge, to date, only a single case with post-stroke aphasia has been reported in which the use of dual tDCS resulted in successful improvement of written language [45]. In this study, the concurrent application of dual-tDCS over the left and right temporo-parietal regions for twelve sessions (three consecutive days for four weeks) combined with sublexical procedure training (i.e., reading and writing lists of syllables) resulted in greater effects of real stimulation compared to sham. Indeed, after dual-tDCS, the patient improved in nonword and word writing, with a generalization effect also in reading [45]. Indeed, recent meta-analyses of neuroimaging studies have reported a considerable overlapping between the cortical regions involved in writing and reading [46,47,48]. In particular, these studies have corroborated the role of several perysilvian cortical areas for gaining access to sublexical procedures, among which is the temporo-parietal cortex [46,48,49,50,51,52]. Accordingly, most tDCS studies have targeted the left temporo-parietal cortex through real stimulation in order to improve word reading efficiency [53] and nonword reading speed [54,55,56]. Interestingly, with reference to the present study, the left temporo-parietal region showed greater activation during nonword with respect to real word writing, suggesting a specific involvement of this region in sublexical processing [48,57].

Thus, in line with all the previous literature, the aim of the present work was to investigate the effect of dual-tDCS over the left and right temporo-parietal cortex combined with a writing treatment in a group of post-stroke chronic aphasia patients with central agraphia. Since all were Italian subjects, we used syllabic segments in order to restore the sublexical route [17,28,29,30].

## 2. Materials and Methods

### 2.1. Participants

Fourteen chronic post-stroke non-fluent aphasics (7 females and 7 males) with a single left hemisphere damage were recruited in the study (see Table 1). Inclusion criteria were native Italian proficiency, a single left hemispheric stroke at least 6 months prior to the investigation, pre-morbid right handedness (based on the “Edinburgh Handedness Questionnaire”; [58]) and no acute or chronic neurological symptoms needing medication. Subjects over 75 years of age and those with seizures, implanted electronic devices (e.g., pacemaker) and previous brain damage were excluded. In order to avoid confounding therapy effects, none of the participants had received language treatment for at least 6 months before the time of inclusion in the study.

### 2.2. Ethics Statement

The data analysed in the current study were collected in accordance with the Helsinki Declaration and the Institutional Review Board of the IRCCS Fondazione Santa Lucia, Rome, Italy. Prior to participation, all patients signed informed consent forms. In particular, they acknowledged that: “The most common reported adverse effects of tDCS in the literature [34] include skin tingling, itching, mild burning sensations, and discomfort, most of which are temporary and well tolerated. The physical adverse effects are restricted to the site of stimulation. The therapist is thoroughly informed as to the technique and adverse effects and the procedure will be fully supervised by a neurologist”. They also knew that: “If you take part in this study, the insurance will cover any possible damage resulting from the application of tDCS”.

### 2.3. Clinical Data

All patients were affected by severe non-fluent aphasia. Subjects were not able to spontaneously speak, but they did not present with articulatory difficulties. To investigate their language performance in depth, each participant underwent a standardized language test (Esame del Linguaggio II; [59]). The test included different language tasks: oral and written noun and verb-naming (n = 20 for noun naming, i.e., penna (pen); n = 10 for verb naming, i.e., mangiare (to eat), dormire (to sleep)), words and sentences repetition, reading and writing under dictation (words, n = 20, i.e., tavolo (table), sentences, n = 10, i.e., il marinaio sale sulla nave (the sailor gets on the ship)), nonword syllable repetition, reading and writing under dictation (n = 20, i.e., bo, fime, tarino), and oral and written word (i.e., pipa (pipe)) and sentence (i.e., apra il libro (open the book)) comprehension. Since the test has been constructed in order to investigate language abilities in severe aphasia, all were high and medium frequency words. The stimuli were divided according to the grammatical class (nouns, verbs), frequency (high ≥ 30/million, medium ≥ 20/million) and length (short = 4/6 phonemes, long ≥ 6 phonemes).

All subjects were able to produce few words in noun and verb naming, and to repeat and to read some words and nonwords (see Table 1). They presented with a very severe impairment in writing. They were not able to write any single words and/or syllables (nonwords), showing severe damage both to the lexical and sublexical procedures [4]. All subjects were able to auditorily comprehend simple words and commands of the language test (Esame del Linguaggio II; [59]), while they were not able to accomplish more complex auditory comprehension tasks (Token test cut-off 29/36 [60]).

### 2.4. Materials

Two lists of sixty stimuli were constructed. Each list contained twenty syllables (e.g., BU, CE, FO), twenty disyllabic (CVCV consonant–vowel, e.g., BUCE) and twenty trisyllabic nonwords (CVCVCV consonant–vowel, e.g., BUCEFO). According to the International Phonetic Alphabet (International Phonetic Association, [61]), syllables encompass different places (e.g., plosive, nasal, fricative) and manners of articulation (e.g., bilabial, dental, velar).

### 2.5. Procedure

#### 2.5.1. Transcranial Direct Current Stimulation (tDCS)

Transcranial direct current stimulation (tDCS) was applied using a battery driven Eldith (neuroConn GmbH) Programmable Direct Current Stimulator with a pair of surface-soaked sponge electrodes (5 cm × 7 cm). Real stimulation consisted of 20 min of 2 mA direct current with the anode placed over the ipsilesional and the cathode over the contralesional temporo-parietal cortex (CP5 and CP4 of the extended International 10–20 system for EEG electrode placement). For sham stimulation, the same electrode positions were used. The current was ramped up to 2 mA and slowly diminished over 30 s to guarantee the typical initial tingling sensation [62]. In both conditions (real vs. sham), patients were administered simultaneous language treatment (see below), which was performed in ten daily one-hour treatment sessions (Monday–Friday, weekend off, Monday–Friday). There was a 14-day intersession interval between the real and the sham condition. The order of conditions was randomized across subjects. Both the clinician and the patient were blinded with respect to the administration of tDCS, which was applied by a third person who was not involved in the study. At the end of each condition, subjects were asked if they were aware of which condition (real or sham) they were in. None of the subjects was able to determine differences in sensation and intensity between the two conditions.

#### 2.5.2. Language Treatment

All patients underwent the standardised language test at the beginning (baseline; T0), at the end (T10) of each treatment condition, and one week after the end of the treatment (follow-up; F/U).

Since each patient showed the presence of severe agraphia which equally affected the lexical and sublexical route [4,5,6] and our patients were all native Italian speakers, based on previous evidence [6,24,63], the intervention was aimed at restoring the sublexical route via syllable repetition, reading and writing.

Before the treatment, all 120 stimuli (syllables, disyllabic and trisyllabic nonwords) were auditorily randomly presented to each patient. The participant had to read and write each stimulus within 30 s. As all participants failed to correctly write all the presented stimuli, the whole lists were subdivided into the two lists of sixty stimuli. Each list was randomly assigned to each participant and to one of the two experimental conditions (real vs. sham). For each condition, the order of presentation of stimuli was randomized across the training sessions. The therapy method was similar for all patients. For each condition (real vs. sham), during each session, the whole list of stimuli was presented. The clinician presented one stimulus at a time and for each stimulus the treatment relied on three different steps which would progressively facilitate the patient in correctly writing it.

Step 1: The clinician auditorily presented the whole stimulus and asked the patient to write it. If the patient correctly wrote the stimulus, the clinician would proceed with the other stimulus, but if he or she made mistakes the clinician would move on to the next step.

Step 2: The clinician auditorily presented the stimulus again and asked the patient to write it. If the patient correctly wrote the stimulus, the clinician would ask the participant to read it, but if he or she made mistakes the clinician would move on to the next step.

Step 3: The clinician wrote the stimulus and asked the patient to read it. After a few seconds, the clinician covered the stimulus and asked the patient to write it again. If the patient could not solve the task, the clinician proceeded with another stimulus.

The response was registered as correct only if the patient wrote the stimulus in the first step. The clinician manually recorded the response type on a separate sheet.

#### 2.5.3. Data Analysis

The patients’ performance was evaluated by considering the mean percentage of response accuracy for syllables and disyllabic and trisyllabic nonwords for each condition (real vs. sham). Data were analysed using SPSS 20.0 software (IBM SPSS Statistics, version 20, Armonk, NY, USA). Three repeated measures ANOVAs were performed separately for syllables and disyllabic and trisyllabic nonwords. For each analysis, two “within” factors were considered: CONDITION (real vs. sham) and TIME (baseline (T0) vs. end of treatment (T10) vs. follow-up (FU)). The post hoc Bonferroni test was conducted on the significant effects observed in the ANOVA. The values of *p* ≤ 0.05 were considered statistically significant. Before and after each treatment condition, the patients’ responses to the different re-administration of the standardized language test (Language Examination II; [59]) were also analysed using *χ*^2^-test.

## 3. Results

### 3.1. Accuracy Data

#### 3.1.1. Syllables

**Figure 1:**
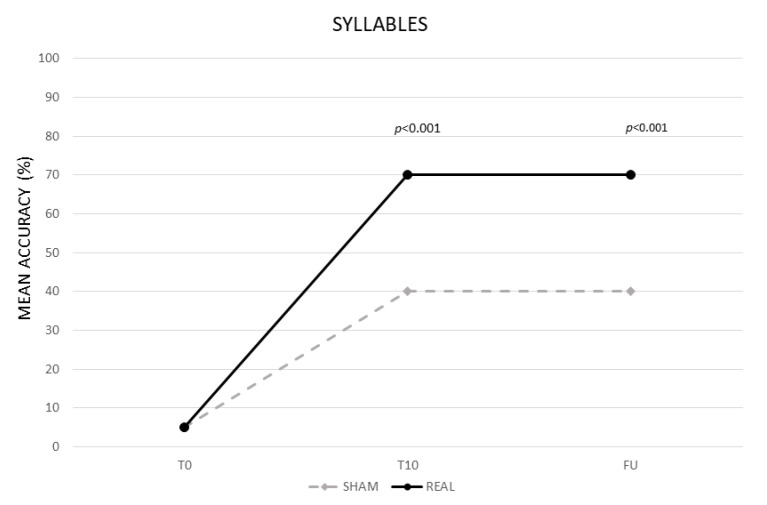
Mean percentage of response accuracy for syllables at baseline (T0), at the end of treatment (T10) and at follow-up (F/U) for the real and sham condition, respectively.

The analysis showed a significant effect of CONDITION (real vs. sham, F (1,13) = 83,27, *p* < 0.001) and TIME (baseline (T0) vs. end of treatment (T10) vs. follow-up (F/U), F (2,26) = 353,78, *p* < 0.001). The interaction TIME × CONDITION was also significant (F (2,26) = 88,65, *p* < 0.001). The Bonferroni’s post hoc test revealed that, while no significant differences emerged in the mean percentage of correct syllables between the two conditions at T0 (real 5% vs. sham 5% *p* = 1), the mean percentage of accuracy was significantly greater in the real than in the sham condition at T10 (real 70% vs. sham 40%, *p* < 0.001) and persisted at F/U (real 70% vs. sham 40% *p* < 0.001). Significant differences also emerged between T0 and T10 for the sham condition (35%, *p* < 0.001) (see Figure 1).

We ran further analysis by adding the order of conditions (real vs. sham) as a fixed factor. The analysis revealed that the results were not significantly affected by the order of condition (F (1,12) = 1.16, *p* = 0.30). Moreover, a mixed analysis of variance (ANOVA) with ORDER of CONDITIONS as the between-subjects factor (first treatment vs. second treatment) and CONDITION (real vs. sham) and TIME (baseline (T0) vs. last day (T10) vs. follow up (FU)) as two within-subjects factors confirmed that the ORDER of CONDITIONS was not significant (F(1,12) = 1.40, *p* = 0.26) as well as the interaction of ORDER of CONDITIONS × CONDITION (F (1,12) = 0.00 *p* = 1), ORDER OF CONDITIONS × TIME (F (2,24) = 0.02, *p* = 0.98) and ORDER of CONDITIONS × CONDITION × TIME (F (2,24) = 1.64, *p* = 0.21). As in the previous analysis, independently of the order of conditions, the interaction of CONDITION × TIME was significant (F (2,24) = 55.30, *p* < 0.001).

#### 3.1.2. Disyllabic Nonwords

**Figure 2:**
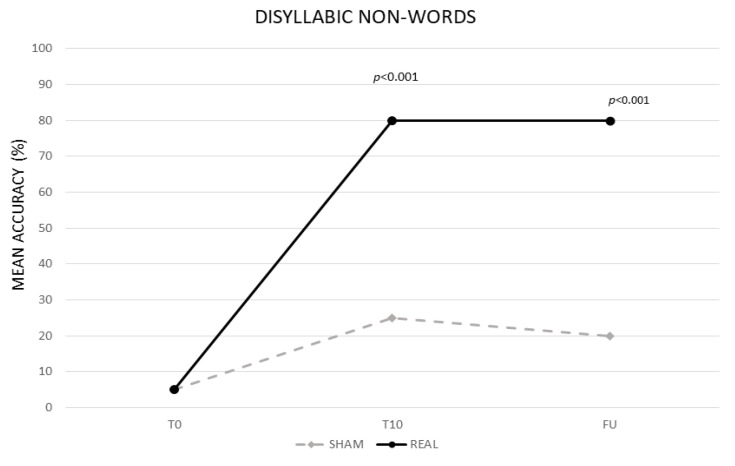
Mean percentage of response accuracy for disyllabic nonwords at baseline (T0), at the end of treatment (T10) and at follow-up (F/U) for the real and sham condition, respectively.

The analysis showed a significant effect of CONDITION (real vs. sham, F (1,13) = 104,99, *p* < 0.001) and TIME (baseline (T0) vs. end of treatment (T10) vs. follow-up (F/U), F (2,26) = 223.08, *p* < 0.001). The interaction TIME × CONDITION was also significant (F (2,26) = 117.19, *p* < 0.001). The Bonferroni’s post hoc test revealed that, while no significant differences emerged in the mean percentage of correct syllables between the two conditions at T0 (real 5% vs. sham 5%, *p* = 1), the mean percentage of accuracy was significantly greater in the real than in the sham condition at T10 (real 80% vs. sham 25%, *p* < 0.001) and persisted at F/U (real 80% vs. sham 20% *p* < 0.001). Significant differences also emerged between T0 and T10 for the sham condition (20%, *p* < 0.001) (see Figure 2).

We ran further analysis by adding the order of conditions (real vs. sham) as a fixed factor. The analysis revealed that the results were not significantly affected by the order of condition (F (1,12) = 1.77, *p* = 0.21). Moreover, a mixed analysis of variance (ANOVA) with ORDER of CONDITIONS as the between-subjects factor (first treatment vs. second treatment) and CONDITION (real vs. sham) and TIME (baseline (T0) vs. last day (T10) vs. follow up (FU)) as two within-subjects factors confirmed that the ORDER of CONDITIONS was not significant (F(1,12) = 3.7, *p* = 0.09) as well as the interaction of ORDER of CONDITIONS × CONDITION (F (1,12) = 0.06, *p* = 0.81), ORDER OF CONDITIONS × TIME (F (2,24) = 0.49, *p* = 0.62) and ORDER of CONDITIONS × CONDITION × TIME (F (2,24) = 0.02, *p* = 0.98). As in the previous analysis, independently of the order of conditions, the interaction of CONDITION × TIME was significant (F (2,24) = 108.32, *p* < 0.001).

#### 3.1.3. Trisyllabic Nonwords

**Figure 3:**
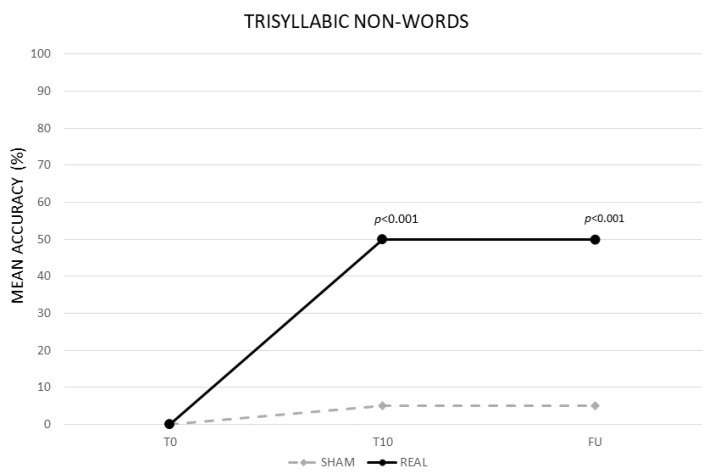
Mean percentage of response accuracy for trisyllabic nonwords at baseline (T0), at the end of treatment (T10) and at follow-up (F/U) for the real and sham condition, respectively.

The analysis showed a significant effect of CONDITION (real vs. sham, F (1,13) = 408.82, *p* < 0.001) and TIME (baseline (T0) vs. end of treatment (T10) vs. follow-up (F/U), F (2,26) = 477.66, *p* < 0.001). The interaction TIME × CONDITION was also significant (F (2,26) = 386.56, *p* < 0.001). The Bonferroni’s post hoc test revealed that, while no significant differences emerged in the mean percentage of correct syllables between the two conditions at T0 (real 0% vs. sham 0%, *p* = 1), the mean percentage of accuracy was significantly greater in the real than in the sham condition at T10 (real 50% vs. sham 5%, *p* ≤ 0.001) and persisted at F/U (real 50% vs. sham 5% *p* < 0.001). No significant differences emerged between T0 and T10 for the sham condition (0%, *p* = 1) (see Figure 3).

We ran further analysis by adding the order of conditions (real vs. sham) as a fixed factor. The analysis revealed that the results were not significantly affected by the order of condition (F (1,12) = 1.000, *p* = 0.34). Moreover, a mixed analysis of variance (ANOVA) with ORDER of CONDITIONS as the between-subjects factor (first treatment vs. second treatment) and CONDITION (real vs. sham) and TIME (baseline (T0) vs. last day (T10) vs. follow up (FU)) as two within-subjects factors confirmed that the ORDER of CONDITIONS was not significant (F(1,12) = 0.09, *p* = 0.77) as well as the interaction of ORDER of CONDITIONS × CONDITION (F (1,12) = 0.44, *p* = 0.52), ORDER OF CONDITIONS × TIME (F (2,24) = 0.61, *p* = 0.55) and ORDER of CONDITIONS × CONDITION × TIME (F (2,24) = 0.43, *p* = 0.66). As in the previous analysis, independently of the order of conditions, the interaction of CONDITION × TIME was significant (F (2,24) = 369.57, *p* < 0.001).

Finally, “generalization effects” in the language test indicated that, in most of the patients, there was a significant difference in the percentage of correct responses before and after the treatment in different language tasks, which was greater after the real than in the sham condition (see Table 2).

## 4. Discussion

The aim of the present study was to explore whether dual-tDCS combined with a language treatment would improve writing skills in fourteen chronic patients with severe agraphia. Since all were Italian patients, we employed as a treatment nonword writing in order to rely on the sublexical procedure [17,28,29,30]. We used dual-tDCS based on the hypothesis that simultaneously up- and down-regulating activity, respectively, in the left and right temporo-parietal cortex would enhance the recovery process in the left hemisphere [35,36,37,64]. Indeed, the comparison between different electrode montages, which have been used in tDCS post-stroke aphasia studies, has shown that bilateral stimulation over the left and right inferior frontal gyrus (IFG) determines a clear incoming current into the left hemisphere more focally distributed over the left perilesional region and a component of outgoing current from the right hemisphere with respect to unilateral montage with the anode placed over the left IFG [35,36,37,64]. Accordingly, our findings showed that, after real stimulation, there was a greater improvement in syllables and disyllabic and trisyllabic nonword writing with respect to the sham condition which persisted after one week from the end of the treatment. This last result is consistent with the previous tDCS literature in healthy subjects and brain-damaged individuals showing longer-term changes in motor abilities, learning and language recovery [36,65,66,67,68,69]. Indeed, unlike single tDCS session effects, which are mediated by transient neural modulations [32], repeated stimulation sessions combined with training are thought to act via mechanisms similar to long-term potentiation, which is critical for neuroplasticity and memory consolidation [66,70,71]. Significant differences were also present in the sham condition, but only for syllabic and disyllabic nonwords. Thus, the language training alone was successful, but only for the simplest stimuli. Interestingly, patients had a better writing performance with disyllabic nonwords with respect to one syllable. Although we do not have a final interpretation of this result, we believe that it could be determined by the partial sparing of nonwords repetition which was already present at the beginning of the treatment in most of the subjects (see Table 1). Indeed, subjects might have made use of spared phonological rehearsal processes which have facilitated the retention of longer nonwords and, thus, their conversion into the corresponding graphemes.

Moreover, most of the patients showed significant changes in different oral and written language tasks of the language test, administered before and after the treatment, particularly in oral and written noun and verb naming, nonword and word writing to dictation and word/nonword reading. Thus, as already suggested by previous studies, relying on the sublexical procedure also resulted in generalization effects of the acquired learning on untrained items [3,6,24,25,26,27,63]. Indeed, recent meta-analyses of neuroimaging studies have shown that the cortical areas involved in written language overlap with those implicated in reading [46,47]. Thus, in agreement with those studies, our results showed that dual temporo-parietal tDCS also exerted its influence on reading. Accordingly, high frequency repetitive transcranial magnetic stimulation (rTMS) over the left inferior parietal lobule (IPL) improves nonword reading accuracy [72]. A similar improvement was also found in dyslexic patients [73] after stimulation of the left IPL and in adults with typical reading after anodal stimulation over the left posterior temporal cortex compared to sham [53]. Several tDCS studies have also suggested that the left temporo-parietal region refers to a large network implicated in phonological processing [54,55] and in the acquisition of new vocabulary [74,75,76,77,78,79]. Indeed, Price [80] has pointed out that the left temporo-parietal cortex is involved in several language processes, including phonological, orthographic and semantic processing and grapheme-to-phoneme conversion. In particular, according to several neuroimaging studies, the temporo-parietal cortex is activated during phonological encoding and memory performance for new words [49,81,82]. In line with this evidence, Savill and collaborators [79] have shown that tDCS over the temporo-parietal cortex facilitated word learning by enhancing the acquisition of phonological forms during a serial word recall task. In addition, Maloney’s work [83] has indicated that nonword letter strings can be easily represented in the orthographic input and phonological output lexicon after a small number of repetitions. Indeed, the authors have shown the development of a new orthographic and phonological lexical route through the conversion from sublexical to lexical procedures for nonwords.

Thus, given the role played by the left temporo-parietal cortex in several language tasks, we should have expected a generalization effect to other language tasks, which was the case.

Before concluding, a final point is worth noting, which we believe is highly relevant from a clinical perspective. Indeed, since all of our patients were in the chronic phase and they had very severe agraphia, the presence of an improvement, after dual-tDCS, for the most difficult items (i.e., trisyllabic nonwords) and generalization effects in the language tasks was not necessarily taken for granted. Previous results on chronic post-stroke aphasia led to similar results [36,37,38], suggesting that the combination of tDCS with language training also boosts the recovery process in cases of severe aphasia. Indeed, tDCS induces neuroplasticity in humans, thus, it has the potential to foster physiological plasticity in neurological diseases such as post-stroke chronic aphasia [84,85,86]. Given that, in the chronic phase, interhemispheric connections between the left and the right hemisphere might be detrimental for language recovery, we might hypothesize that, in our work, dual tDCS has temporarily reversed the interhemispheric imbalance, thus improving language skills in our patients [87,88].

In conclusion, although our results are encouraging for identifying tDCS protocols for language improvement in chronic post-stroke aphasia, we are aware that they have some limitations due to the small sample size considered and the absence of pre-treatment mapping of spared language regions using functional MRI. Indeed, we know that the choice of our stimulation sites might have been not entirely appropriate due to variable lesion sizes and locations among our patients as well as interindividual differences in functional language network reorganization. Thus, fMRI would have helped us to better identify the stimulation sites.

However, apart from these limitations, we believe that research concerning tDCS in aphasia is crucial to promote our understanding of the neural mechanisms by which tDCS improves language functions in the chronic stage.

## Figures and Tables

**Table 1:** Sociodemographic and clinical data of the fourteen non-fluent aphasic patients. [59]. Legend: *p* = Participants; Ed. Level = Educational Level; I = Ischaemic; H = Haemorrhagic; LH = Left hemisphere; FTI = fronto-temporo-insular; T = temporal; FTP = fronto-temporo-parietal; Oral NN = Noun Naming; Oral VN = Verb Naming; Written NN = Noun Naming; Written VN = Verb Naming; WR = Word Repetition; NWR = Nonword Repetition; W Read = Word Reading; NW Read = Nonword Reading; WD = Word under Dictation; NWD = Nonword under Dictation; TT = Token Test (cut-off score 29/36).

*p*	Sex	Age	Ed. Level	Time Post Onset	Stroke Type	Lesion Side LH	Oral NN	Oral VN	Written NN	Written VN	W R	NWR	W Read	NW Read	WD	NW D	TT
1	M	57	13	3 years	I	FTI	7.5	10	0	0	35	22.5	15	5	0	0	4
2	M	59	13	3 years	I	T	0	0	0	0	25	30	17.5	15	0	0	2.5
3	M	53	17	1 year	I	FTI	0	0	0	0	22.5	30	12.5	15	0	0	6
4	F	65	8	3 years	I	FTI	15	15	0	0	42.5	20	20	10	0	0	10
5	M	55	13	4 years	I	T	15	10	0	0	20	15	15	5	0	0	10
6	M	64	13	1 year	I	FTP	0	0	0	0	0	0	0	0	0	0	2
7	M	62	17	1 year	I	FTI	7.5	0	0	0	40	32.5	12.5	2.5	0	0	10
8	M	63	17	4 years	I	FTI	0	0	0	0	0	0	0	0	0	0	2
9	F	55	13	3 years	I	FTP	15	15	0	0	80	35	32.5	10	0	0	8
10	F	57	8	1 year	I	T	0	0	0	0	0	0	0	0	0	0	10
11	F	55	13	1 year	H	FTP	15	15	0	0	85	30	15	15	0	0	10
12	F	65	13	2 years	I	FTP	10	15	0	0	15	10	15	15	0	0	10
13	F	58	8	4 years	I	FTP	10	10	0	0	20	20	12.5	5	0	0	7.5
14	F	65	13	3 years	I	FTP	15	12.5	0	0	10	15	15	5	0	0	4

**Table 2:** Percentage of correct responses in the different language tasks (Esame del Linguaggio II, [59]) at baseline (T0) and at the end of treatment (T10) for the real and sham condition, respectively (cut-off score 100%).

*p*	C	Oral NN		Oral VN		Written NN		Written VN		W R		Ntable R		W Read		NW Read		W Dict		NW Dict	
		T0	T10	T0	T10	T0	T10	T0	T10	T0	T10	T0	T10	T0	T10	T0	T10	T0	T10	T0	T10
**Real First**																					
1	R	7.5	80 ^	10	60 ^	0	55 ^	0	40 ^	35	92.5 ^	22.5	90 ^	15	67.5 ^	5	50 ^	0	72.5 ^	0	55 ^
	S	80	85	60	70	55	55	40	45	92.5	90	90	95	67.5	70	50	45	72.5	75	55	55
3	R	0	67.5 ^	0	60 ^	0	37.5 ^	0	35 ^	22.5	85 ^	30	80 ^	12.5	42.5 ^	15	60 ^	0	50 ^	0	52.5 ^
	S	67.5	80 *	60	80 **	37.5	35	35	40	85	92.5	80	95 **	42.5	45	60	60	50	55	52.5	62.5
5	R	15	70 ^	10	80 ^	0	42.5 ^	0	32.5 ^	20	82.5 ^	15	80 ^	15	60 ^	5	62.5 ^	0	57.5 ^	0	67.5 ^
	S	70	77.5	80	85	42.5	42.5	32.5	35	82.5	97.5 ^	80	80	60	60	62.5	62.5	57.5	57.5	67.5	52.5
7	R	7.5	55 ^	0	60 ^	0	30 ^	0	27.5 ^	40	72.5 ^	32.5	87.5 ^	12.5	62.5 ^	2.5	47.5 ^	0	40 ^	0	45 ^
	S	55	75 **	60	70	30	35	27.5	27.5	72.5	72.5	87.5	87.5	62.5	57.5	47.5	47.5	40	40	45	45
9	R	15	45 ^	15	65 ^	0	40 ^	0	15 ^	80	100 ^	35	82.5 ^	32.5	80 ^	10	50 ^	0	50 ^	0	12.5 ^
	S	45	54	65	70	40	55 *	15	15	100	92.5	82.5	92.5 *	80	80	50	50	50	42.5	12.5	17
11	R	15	60 ^	15	80 ^	0	55.5 ^	0	17.5 ^	85	95 *	30	92.5 ^	15	60 ^	15	77.5 ^	0	65 ^	0	20 ^
	S	60	60	80	80	55.5	60	17.5	20	95	97.5	92.5	95	60	60	77.5	82	65	65	20	27.5
13	R	10	67.5 ^	10	75 ^	0	42.5 ^	0	30 ^	20	65 ^	20	70 ^	12.5	65 ^	5	45 ^	0	55 ^	0	47.5 ^
	S	67.5	65	75	80	42.5	50	30	30	65	72.5	70	70	65	65	45	50	55	57.5	47.5	47
**Sham First**																					
2	S	0	20 ^	0	5	0	10 **	0	5	25	35	30	35	17.5	12.5	15	15	0	20 ^	0	5
	R	20	45 ^	5	10	10	40 ^	5	20 **	35	85 ^	35	90 ^	12.5	57.5 ^	15	57.5 ^	20	50 ^	5	42.5 ^
4	S	15	27.5 *	15	30 *	0	5	0	10 **	42.5	47.5	20	20	20	30	10	10	0	25 ^	0	25 ^
	R	27.5	55 ^	30	62.5 ^	5	50 ^	10	35 ^	47.5	62.5 *	20	62.5 ^	30	75 ^	10	62.5 ^	25	55 ^	25	67.5 ^
6	S	0	2.5	0	0	0	0	0	0	0	17.5 ^	0	20 ^	0	0	0	5	0	0	0	5
	R	2.5	15 **	0	0	0	10 **	0	0	17.5	47.5 ^	20	65 ^	0	10 **	5	15 *	0	0	5	25 ^
8	S	0	0	0	0	0	0	0	0	0	5	0	5	0	0	0	0	0	0	0	0
	R	0	20 ^	0	0	0	15 ^	0	0	5	17.5 **	5	25 ^	0	12.5 ^	0	15 ^	0	0	0	20 ^
10	S	0	0	0	0	0	5	0	0	0	20 ^	0	20 ^	0	15 ^	0	2.5	0	0	0	0
	R	0	45 ^	0	35 ^	5	35 ^	0	10 **	20	65 ^	20	62.5 ^	15	42.5 ^	2.5	25 ^	0	40 ^	0	35 ^
12	S	10	15	15	20	0	10 **	0	0	15	20	10	22.5 *	15	30 *	15	20	0	25 ^	0	10 **
	R	15	60 ^	20	65 ^	10	55 ^	0	15 ^	20	72.5 ^	22.5	75 ^	30	75 ^	20	65 ^	25	70 ^	10	50 ^
14	S	15	32.5 **	12.5	20	0	5	0	0	10	17.5	15	15	15	27.5 *	5	10	0	15 ^	0	5
	R	32.5	80 ^	20	80 ^	5	45.5 ^	0	20 ^	17.5	72.5 ^	15	65 ^	27.5	75 ^	10	35 ^	15	65 ^	5	57.5 ^

Legend: *p* = Participants; C = Conditions; Oral NN = Noun Naming; Oral VN = Verb Naming; Written NN = Noun Naming; Written VN = Verb Naming; WR = Word Repetition; NWR = Nonword Repetition; W/NW Read = Word/Nonword Reading; W/NW Dict = Word/Nonword under Dictation; S = Sham; R = Real stimulation; * = *p* < 0.05; ** = *p* < 0.01; ^ = *p* < 0.001.

## Data Availability

The data presented in this study are available on request from the corresponding author. The data are not publicly available due to ethical and privacy restrictions.
